# Additional Notes on Provincial Asylums for the Insane in France; with a Brief Report of the Institution at Illnau, in the Grand Duchy of Baden

**Published:** 1852-04-01

**Authors:** John Webster

**Affiliations:** FELLOW OF THE ROYAL COLLEGE OF PHYSICIANS; CONSULTING PHYSICIAN TO ST. GEORGE AND ST. JAMES'S DISPENSARY, ETC.


### Original Communications.

### ADDITIONAL NOTES ON PROVINCIAL ASYLUMS FOR THE INSANE IN FRANCE; WITH A BRIEF REPORT OF THE INSTITUTION AT ILLNAU, IN THE GRAND DUCHY OF BADEN.

BY JOHN WEBSTER, M.D., F.R.S., FELLOW OF THE ROYAL COLLEGE OF PHYSICIANS; CONSULTING PHYSICIAN TO ST. GEORGE AND ST. JAMES'S DISPENSARY, ETC.

(*Continued from page* 139.)

#### FAINS ASYLUM.

THIS public institution for lunatics is agreeably situated in the valley of the Ornain, about two miles from Bar le Duc, the chief town of the Meuse department. The surrounding hills—lying at a short distance—are partially wooded, and form a kind of amphitheatre; but, although the asylum has been built on low ground, which occasionally becomes damp, the situation seems open, well-ventilated, and is not considered insalubrious. The building has nearly the figure of an H, being originally constructed by Napoleon as a barrack, but afterwards it became a mendicity depôt, till 1836, when its chief destination was changed to a receptacle for the insane. Since that period to the present, the house has been almost exclusively appropriated to such purposes: the exceptions being in reference to indigent persons affected with skin diseases, syphilis, or scrofula, who may be admitted by an order from the Préfet. However, although the number of these inmates seldom exceeds twenty or twenty-five, the practice is so objectionable that it ought to be altogether abolished.

The medical staff attached to this institution consists of one physician—Dr. Fornasari, and an interne. Both officers will soon be resident, whenever the apartments destined for the former gentleman are completed. In the interim, he resides at Bar le Duc, but visits the asylum every forenoon, or oftener, when necessary. Dr. Fornasari labours most assiduously in the discharge of his professional duties; and being ably seconded by his intelligent interne—M. Berrut, the patients receive every attention which their cases require. Formerly, the offices of physician and director were filled by one individual; but the number of inmates having considerably increased during recent years, the responsibilities became too onerous for a single official, hence, both duties were separated. In proof of this inference, it may be stated that, the total patients under treatment in 1845 were 271; whereas, during 1848 they had augmented to 343 inmates, which has ever since continued about the average number. On the day of my visit to Fains the insane residents amounted to 341; of whom 186 were male, and 155 female patients. Besides the above, twenty-one persons affected with cutaneous diseases, by syphilis, or scrofula, also resided in the house; eleven being males and ten females. Consequently, 362 patients altogether were then under the physician's superintendence.

Amongst the 341 insane persons now reported, about one-fifth, or seventy patients, were considered curable. The epileptics amounted to thirty; including twenty-one male and nine female patients. Those affected with general paralysis were fifteen, comprising fourteen men, but only one woman; whilst the total number of dirty patients was forty-five, or nearly one-eighth of the entire population, and consisted of nineteen men with twenty-six women. This aggregate amount was, however, less than on the 31st of last December, when sixty were similarly classified, out of 379 inmates then under treatment; hence, giving about seventeen such cases in every hundred lunatics. Besides the above details, it should be also mentioned that, all were not indigent persons, but included private patients from various districts; the actual number of the latter class being fifty-nine individuals, of whom thirty-one were men and twenty-eight women, who paid from 500 to 1800 francs per annum; although one lady of rank was charged considerably higher, in consequence of the superior accommodation afforded.

Notwithstanding this institution is appropriated for indigent insane patients chargeable to the Meuse department, some are natives of other localities. Thus, about one-fourth, or eighty-five inmates, belonged to the department of the Seine, having been transferred from Paris to this asylum, to relieve the overcrowded metropolitan establishments. Many of the above cases being of long continuance, and nearly hopeless of future improvement, readily explains why the proportion of curable patients is so inconsiderable, compared with the large amount of inmates of an opposite category. This feature seems, however, by no means peculiar to the asylum at Fains, being frequently observed in other public institutions for the insane; whilst such facts sufficiently account for the limited number of cures reported. Hence, it cannot be surprising, should the ratio of deaths considerably exceed the amount which might be reasonably anticipated, were patients admitted during the early stages of their mental disease; when there exists a far greater hope of amelioration. So much valuable time is frequently lost before lunatics are sent to an asylum that, attacks of mania, which might have been speedily cured, or materially alleviated, by judicious treatment then instituted, were thereby unfortunately rendered chronic, if not ultimately irremediable. Through such dilatory and blameable proceedings, the afflicted party thus becomes a permanent source of anxiety to relatives; and, if in indigent circumstances, consequently entails considerable annual expense to the commune, when sent to a departmental asylum.

On the day I inspected the Fains institution, only one man and two women were under restraint; besides a third female, who was merely confined in demi-camisole. It ought, however, to be mentioned in explanation that, the individual reported as the solitary male inmate under confinement, was merely tied slightly to his bed, in order to prevent accidents, in consequence of being totally helpless. Dr. Fornasari declared himself a strong opponent of restraint; and said he never used the strait-waistcoat, unless its application became absolutely necessary, to prevent lunatics from inflicting injury on others or themselves. It ought further to be stated, when he first became attached to this asylum, several patients were then confined by camisoles, as they seemed very violent, and therefore considered dangerous. Nevertheless, having been soon freed from restraint, whilst pickaxes and wheelbarrows were substituted, most of these inmates went quietly to work in the gardens. This proceeding produced excellent effects, without ever causing any reason to regret its adoption; seeing insane patients thus treated become in a short time tranquil, well-conducted, and laborious. An interesting example of the beneficial effects thus produced, contrasted with the ancient method of treating lunatics, occurred in a maniac aged twenty-three, who was admitted during 1844 to the Fains Asylum. For a long period afterwards, the arms of this patient were constantly confined by a camisole, which was seldom or ever relaxed, even at meals. Having first examined the lunatic's condition most carefully, Dr. Fornasari ordered all restraint to be removed, and then sent him to dig in the garden, accompanied by an attendant. This poor fellow immediately began to work quietly, and with much ardour, which he continued next day, and subsequently. Afterwards, his mental condition became sensibly improved; and although ultimate convalescence cannot be considered very probable, there soon appeared some prospect of durable improvement.

During 1850 the movement of insane patients reported at this institution is detailed in the subjoined official statement:—

| | Males. | Females. | Total. |
|---|---|---|---|
| Admitted . . . . . . . . | 36 | 24 | 60 |
| Discharged Cured . . . . | 15 | 9 | 24 |
| Died . . . . . . . . . . | 22 | 8 | 30 |

Amongst the twenty-four patients cured, the largest proportion occurred when warm weather prevailed; fourteen individuals having been discharged convalescent, in June, July, and August, whilst only one patient actually recovered during the cold months of November, December, and January. Again, respecting the particular season when mortality proved highest, it is instructive to mention that five deaths, out of the thirty recorded, took place in the summer quarter; whereas, eleven, or more than one-third of the entire number, were reported in November, December, and January. According to such data, the ratio of recoveries was decidedly greatest in warm weather, the reverse being noticed during the cold season. On the other hand, the deaths were fewest in summer, but most numerous in winter. Lastly, amongst the whole thirty fatal cases reported, eleven patients were affected with dementia, six being men and five women; while thirteen, or nearly half the whole deaths, arose from paralysis, all being male patients, but without even one female; since, they are very rarely attacked by this almost incurable form of mental disease.

The bodies of insane patients, in this asylum, being carefully examined after death, I am hence enabled to state that, last year, seventeen autopsies exhibited disorganization—more or less visible—of the brain and nervous system; in five the thoracic organs were chiefly affected; five deaths arose from disease of the abdominal viscera; one by cancer; another from a tumour; and there was one suicide. According to these official statements, it appears that in every fatal case, the patient laboured under serious bodily disease, the largest proportion being affected with paralysis; besides which, one of the examples, although reported to have died from pneumonia, was an epileptic patient, who sunk during an accession of the latter malady. Seeing the proportion of deaths amongst male patients was nearly treble in number, compared with females, it ought to be observed, as explanatory of such results that, the discrepancy chiefly depended upon apoplexy and dementia with paralysis having proved exceedingly fatal to male patients, thirteen cases being recorded by the above two diseases. Respecting the greater frequency of the latter mental malady amongst men than women, doubtless, it arose from the more dissipated habits and excesses of the former sex, especially the abuse of intoxicating drinks, which, unfortunately, prevails in this district.

Again, regarding the age of those patients who died, it also deserves special mention that, only two were under thirty years; whilst the most fatal period amongst men appeared to be from the age of fifty-one to sixty, seeing six of the whole twenty-two fatal cases now reported in that sex actually occurred. Several individuals had, however, attained a more advanced period of life prior to the termination of their mental malady. Thus, three men and two women ranged from sixty-one to seventy when they died, whilst three men and two women were from seventy-one to eighty on the occurrence of that event; thereby showing mania is by no means incompatible with longevity. To which it may be added, as a general remark that, youth favours recovery in recent attacks, but in old age the result seems otherwise.

Before bringing to a close this brief notice of the chief occurrences recorded at the Fains Asylum during 1850, I would further observe, amongst the insane inmates admitted throughout that year, thirteen of the thirty-six male patients laboured under acute mania, six suffered from dementia, six showed symptoms of paralysis complicated with dementia, six were imbeciles, two monomaniacs, one was epileptic, and one idiotic, whilst the remaining case laboured under lypemania. Of the twenty-four female lunatics likewise admitted during last year, eleven had acute mania, seven lypemania, three dementia, while the residuary three patients were all monomaniacs; whereby it appeared no female then received was affected by epilepsy or paralysis. According to these details, acute mania consequently formed the most common variety of mental disease affecting persons recently admitted into this departmental asylum. It farther deserves notice, amongst the twenty-four male and female patients labouring under acute mania at the period of their admission in 1850, thirteen were discharged cured, within the same year; six left considerably ameliorated; and two died; whilst three remained under treatment on the 1st of last January.

Another point in reference to the total cures reported deserves mention; namely, although a large proportion, or ten out of the twenty-four patients discharged convalescent, left the asylum after being under treatment from six weeks to three months, and five from that period to nine months, still other five male patients were also discharged perfectly sane, after having remained not less than six years in the establishment, besides one who had been an inmate since 1840. Notwithstanding these instructive facts conclusively demonstrate mental diseases to be curable in a higher ratio, where an attack is recent, and appropriate treatment employed during the early stages, nevertheless, chronic cases of long standing are not always hopeless of amelioration, nor even sometimes of complete convalescence.

Having no farm attached to this asylum, patients employed in out-door occupations generally labour in the extensive and well arranged gardens adjoining; where many were actually occupied at the time of my perambulations. Other inmates, also, appeared busy in cutting and storing firewood for the ensuing winter's consumption. Some were likewise occupied in the stable and dairy; whilst another party exhibited great zeal in constructing a new summer house for patients who desired shade, or required repose. Various handicraftsmen were besides employed, such as carpenters, weavers, tailors, and so forth; Dr. Fornasari being of opinion that, bodily exertion is indispensable in the management of insanity; consequently, every means available to promote that essential object are put in requisition; seeing labour, skilfully applied, proves morally and physically hygienic to every living being, but especially to the insane. Its judicious application ought, therefore, to be always advocated when combined with nutritious food and proper regimen.

In the treatment of female patients in this asylum, similar principles also seemed the guiding rules of conduct; many women being occupied in making or mending clothes, washing, cooking, cleaning apartments, and in various household duties. In consequence of numerous lunatics being thus busily employed, the general aspect of both divisions appeared tranquil, especially the female wards; and as the bodily health, generally speaking, of most inmates was satisfactory, considering their mental afflictions, the impressions produced respecting the Fains establishment, as also my subsequent recollections, were favourable, particularly of its medical superintendence.

Although space does not permit entering at any length upon the medical treatment pursued in this, as in other French asylums, nevertheless, Dr. Fornasari having recently paid much attention to epilepsy, and the treatment considered best adapted for that terrible disease, it may be interesting to state the result of his experience in reference to the valerianate of zinc, which he has recently employed in eight epileptic men and four women, of whom none were either paralysed or imbecile. Through this metallic remedy, the attacks of epilepsy appeared to be diminished both in frequency and intensity. Indeed, several of the above twelve patients who actually suffered from seizures every three, six, eight or ten days, previous to taking valerianate of zinc, derived so much benefit that, he reported more than three months had elapsed without any recurrence; and when a fit did supervene, the attack lost much of its previous intensity and violence; whilst the physical health of every patient so treated, with one exception, became generally ameliorated. The remedy was given at first in moderate doses, but afterwards gradually augmented; and in some cases, even to the extent of three grains and a half every morning, fasting. Besides an occasional purgative, frequent baths were likewise employed, and the diet regulated carefully. As epilepsy is almost incurable, especially in advanced life, or even in middle age, and being very little amenable to remedies, the experience of Dr. Fornasari, in regard to valerianate of zinc in that disease, becomes important, and seems sufficiently encouraging to deserve farther trial by practical physicians.

When several improvements now contemplated, with those actually in progress are completed, a more minute classification of the inmates will be established than is at present possible, owing to the crowded state of various dormitories. Besides new workshops, upon an extensive scale, a large bath-house has been decided upon, and will be forthwith commenced. The wooden bedsteads now in general use are also being replaced by others made of iron; so that here, as in many other departmental asylums, it may be justly said, the managing authorities, instead of falling asleep at their posts, are zealous in remedying ancient defects, and in improving the accommodation afforded to residents, in order to relieve the afflictions of those unfortunate human beings committed to their care and keeping. One article in the dormitories of Fains attracted my attention, from being the first occasion in which I had ever noticed a similar appendage to any French asylum previously visited. Besides the ordinary bedclothes, each patient was provided with a large eider down pillow—the "plumeau" in France, or "eider decke" of Germany—placed over the counterpane. This custom reminded me of "Deutschland;" and indicated that here, as in Lorraine, but especially Alsace, the inhabitants still retain considerable resemblance to the race from whence many of their progenitors originally sprung. Nay, the latter province seems more allied to Germany than France, by language, blood, physical appearance, and temperament of its natives, or geographical position. Nevertheless, politically speaking, the general population here consider themselves more truly French and republican, than persons belonging to many other districts, who are, viewed geographically, as much entitled to assume these distinctive denominations.

Before concluding my report respecting the Fains asylum, in order to illustrate the attention now paid throughout France, to give greater protection to numerous but formerly often neglected lunatics, it may interest readers if a brief summary was added, showing the general movement of insane patients under medical care at this institution, during a period of six years, ending on the 31st of December, 1850. According to official documents, 496 lunatics, including both sexes, were admitted from the first of January, 1845, to the end of last December; and 118 were discharged cured; thus giving 23·99 per cent. recoveries, if compared with the total admissions. On the other hand, the deaths being altogether 219, the rate of mortality therefore amounted to 44·15 per cent., similarly calculated. However, as many of the lunatics received into this asylum, during 1845 up to 1848 inclusive, comprised incurable men sent from Bicêtre, besides females from the Salpêtrière, most of whom had been long insane; such facts must be always taken into calculation, when estimating both the limited proportion of cures effected, and likewise the amount of deaths; the latter being nearly double the ratio of cases discharged convalescent. No case of cholera having prevailed during 1849, that feature must not be disregarded, in reference to the gross mortality now reported, especially, seeing fewer deaths occurred at the institution during that year, than throughout any similar twelve months, excepting in 1848, when twenty-four patients died from all causes; the number being forty-nine in 1846, fifty-one in 1847, twenty-six in 1849, and thirty during 1850, as previously mentioned. Believing this immunity from epidemic cholera—which elsewhere proved frequently fatal—was greatly promoted through augmented attention then paid to hygienic measures, to better dietary, and the employment of other means recommended by medical authority, their adoption consequently reflects much credit upon the managing committee of the Fains asylum.

### AUXERRE ASYLUM.

Having felt it imperative, as an impartial reporter, to state every circumstance which came under cognizance during both my recent visits to French provincial asylums, perhaps some of the statements recorded may not altogether prove agreeable to parties connected with several of the institutions then inspected. Being always received with much kindness, and having experienced every facility at the respective offices for obtaining information, I nevertheless hope the criticisms expressed in the present, as in my former narrative, will be viewed in the way proposed—namely, to illustrate principles and the practice pursued in public establishments, not to disparage individuals. To make any remarks of the latter description was never intended; and it always produced more pleasure when able to speak in terms of commendation, than to notice matters requiring amendment. On many points English physicians—interested in the management of asylums, and the treatment of lunatics—may derive improvement, as also knowledge from their brethren "d'outre-mer:" although the latter would equally benefit by adopting some of the principles guiding psychological practitioners of this country. In France, Pinel first pointed the way, Esquirol then directed public opinion; whilst in England, Gardiner Hill, Conolly, and other distinguished men followed, nay, have even surpassed former advocates of the new system. Indubitably, more personal coercion prevails, when treating lunatics throughout foreign countries, than in Great Britain; but every year further progress is there made in the right direction. This important practical fact seems apparent in various recently constructed departmental institutions, especially at the establishment about to be described, which constitutes a remarkable and very gratifying illustration regarding the disuse of physical restraint.

Amongst the numerous lunatic institutions I have visited in France, either previously, or during last autumn, the asylum at Auxerre, although equalled in some respects by several departmental establishments, nevertheless, deserves the highest commendations, as it stands out pre-eminent in one important feature, compared with every other similar institution passed under review. However, as this seems anticipating a conclusion respecting which more will be said hereafter, I therefore now proceed, in the manner hitherto adopted, first to narrate the various data upon which inferences may be justly founded, and after giving the entire statement then to express my own opinion.

This public institution for lunatics is contiguous to Auxerre, the capital of the Yonne department. The town contains, at present, nearly 13,000 inhabitants, and formed part of the ancient dukedom of Burgundy. The situation of the asylum is agreeable, and on rather elevated ground, which gently slopes towards the river Yonne. It possesses an extensive yet beautiful prospect of the surrounding district. Auxerre, with its venerable cathedral and elegant spires, being on one side, with vine clad hills ornamented by pretty looking villages in front, but at a distance, whilst the country, in other quarters, consists of corn-fields or vineyards. Hence, the varied yet extensive prospect thus afforded forms altogether a splendid panorama. Contrasted with other departmental lunatic institutions, the one now described, although adjoining a populous neighbourhood, and close to the high road leading to Paris, possesses more local advantages, in my estimation, than any other asylum alluded to throughout these pages, whether in reference to situation or convenience; and if official reports, with actual appearances, may be trusted, doubts cannot exist respecting its salubrity.

Formerly, the Auxerre asylum was a mendicity depôt for disabled and infirm persons of the district. Since 1839, it has become a lunatic institution for indigent insane patients belonging to the Yonne department. The present medical staff consists of one physician—Dr. Girard de Cailleux, and one resident interne; although it is expected there will soon be two of these very useful, nay indispensable, medical officers in all establishments where insane persons are congregated. At the period of my visit, the total resident lunatics amounted to 266—consisting of 109 male and 157 female patients; thus giving a considerable preponderance to the latter sex. Amongst the 266 inmates now enumerated, so large a proportion as 100—including 30 men and 70 women—were pensioners, who paid from 450 to 2400 francs per annum for board and treatment. Taking the whole insane population into account, it was stated, in reply to my inquiry that, paralysis affected 11 cases, of whom six were males and five females. The epileptics amounted to 26, comprising 12 male and 14 female inmates; whilst only four were dirty patients, one being a man and three women. Many of the residents were incurable, as in most public insane establishments both of France and of England; whereby, the cases discharged convalescent are often inconsiderable.

Although mental diseases may not be so frequent amongst the general population of this district of France, as mania has been ascertained to prevail throughout other parts of the republic; and still more, if the ratio be compared with that reported from various northern regions of Europe, the peculiarity, however, becomes instructive which Dr. Girard pointed out, in one of his recent valuable publications, respecting the marked discrepancy in the amount of insanity prevailing throughout different, but even neighbouring portions of the Yonne department. For instance, in the arrondissement of Auxerre, he reports one person to be insane for every 989 inhabitants; whereas, in that of Tonnerre which adjoins, the ratio is one lunatic amongst every 1947 residents; hence giving only half the former proportion.

During 1850, the movement of patients at the Auxerre asylum was reported as under:—

| | Males. | Females. | Total. |
|---|---|---|---|
| Admitted . . . . . . . | 36 | 39 | 75 |
| Discharged Cured . . . | 11 | 11 | 22 |
| Died . . . . . . . . . | 15 | 10 | 25 |

According to the register of autopsies, which is carefully kept at this asylum, in a large proportion of fatal cases, disease of the brain and nervous system was observed, whilst few patients showed either pectoral or abdominal changes of structure. Thus, in 17 of the 25 deaths reported, the brain or its appendages were decidedly disorganized; in three, the organs of the chest exhibited morbid alteration; in four, the abdominal viscera appeared chiefly affected; whilst one case was a suicide.

Having endeavoured, in all previous reports, to give accurate information relative to the amount of restraint employed on the day I visited every institution, it gives me much gratification to state that, in the departmental asylum of the Yonne, *no insane patient, either male or female, was then confined by camisoles;* the return of personal coercion being actually, *Nil.* A couple of male lunatics were, however, placed under temporary seclusion in cells, from being very excited or violent at the time, but both were physically free and unrestrained. Besides these, two insane women were similarly situated, one of whom had also a prolonged tepid bath, with capillary irrigation upon her head; which treatment Dr. Girard often finds very efficacious in tranquillizing furious maniacs.

During our visit to this afflicted inmate she talked incessantly, paid at first no regard to the inquiries made respecting her condition, even would not look at any bystander, and seemed quite bewildered or unconscious of external objects. Instead of speaking in his usual voice, Dr. Girard put some questions in a low whisper, to which the patient at first gave no attention. However, the inquiries being repeated slowly several times, and in a tone nearly inaudible, whilst the physician moved his lips rather more than in ordinary speaking, besides looking steadfastly at the patient's countenance, attention was at last attracted, and she replied distinctly to different questions. The dialogue being continued a little longer, this maniac became much more calm, and ultimately remained tranquil. Referring to the above case, Dr. Girard subsequently said, he generally found similar results were more frequently produced by speaking slowly, and in a subdued tone of voice, instead of addressing similar patients in the ordinary manner. The illustration thus given certainly appeared strong evidence of its efficacy; as some hours afterwards, this inmate was recognised in one of the work-rooms, conducting herself like many of her companions, and without manifesting any symptom of having so recently laboured under previous excitement.

The facts now detailed, with others which were also communicated by Dr. Girard, indicate sufficiently the judicious system usually pursued at this admirably regulated institution; where, occupying insane patients and eschewing personal restraint are the great leading principles of management. Previous to the appointment of the present resident physician-director, now ten years ago, matters were indeed very different, if contrasted with recent appearances. Formerly, although only 140 patients occupied the asylum, there were 56 solitary cells to confine furious maniacs, all having stone walls, and iron bars in every window.

Notwithstanding the large number of cells formerly constructed, it was even proposed to add 22 additional apartments of a similar kind; but Dr. Girard—then in office—having strongly opposed the proposition, the idea was abandoned. Now, these ancient prison-like dens are being all demolished, in order to be replaced by ten new rooms of a very different and superior description: five for male, and also five for female patients. The plan selected is that of a fan; and each cell will have a small court-yard or garden behind, with two opposite entrances. By this means, an attendant placed in the focus, as it may be styled, of either building, can at once inspect the interior of each division, and so overlook every occupant, without being recognised.

As an explanation of the large number of cells formerly existing in this asylum, and of the proposal made some years ago, to construct others in addition, it should be mentioned that, no medical officer actually resided within the establishment, seeing patients were merely visited by a physician practising in Auxerre, who attended during six months, when another practitioner succeeded. Hence, there could exist no efficient medical superintendence; especially as the chief management and entire responsibility were vested in a directrice belonging to a religious order, who lived on the premises, and wielded great authority; indeed, this functionary was endued with power supreme. No system could be more objectionable in a lunatic institution, than the method formerly pursued; which ample experience has conclusively proved to be wholly erroneous. Fortunately, Dr. Girard then became the local presiding genius; and subsequent results have demonstrated the change was in every way judicious.

Besides the evidence already detailed indicating a constant desire to avoid employing personal coercion, several additional cases might be mentioned, as they supply instructive illustrations. Thus, three female patients were particularly pointed out for my observation, who had been described on their entrance, according to reports transmitted from another asylum, as very violent and dangerous lunatics. Nevertheless, the camisoles they wore, when brought to Auxerre, were removed soon afterwards. A soothing method of treatment having been substituted, so much amendment followed that, personal restraint became no longer necessary in any of the patients, therefore, it was wholly abandoned. No iron bars appeared in the windows anywhere; the new courtyards seemed spacious, airy, and were ornamented with trees or shrubbery; whilst covered galleries, open at the sides, and adjoining the new dormitories, had been constructed, which the inmates occupied as work places in fine, or promenaded during bad weather. In the division for agitated patients remarkable tranquillity also prevailed; and, I would add, throughout the entire establishment, every effort was made by the executive to avoid any appearance of a prison, whereby it looked like a workhouse or ordinary hospital.

To occupy patients at various employments is the constant object pursued at this institution. Consequently, a large proportion of inmates are always busy in manual or mental labour. The men as bakers, gardeners, and in handicrafts; besides many actually employed as labourers, to assist the hired workmen who were constructing various new buildings. A number of women, likewise, were engaged in different kinds of female occupations, or in ordinary household employments; hence, throughout the asylum, scarcely any person seemed idle, whilst an appearance of activity reigned in almost every part of the establishment. Besides employing lunatics in judicious bodily labour, it is always considered a great object with Dr. Girard to vary their employments, so as to fix the patient's attention on different objects successively, and thereby improve the individual mental condition, without inducing either fatigue or indifference, which otherwise follow long continued application to one subject consecutively. Music and singing also constitute important adjuvants to the various recreations employed, in addition to medical or ordinary remedial measures. Dr. Girard is a strenuous advocate for music in the treatment of mental diseases; and speaking generally from his experience, entertains decided views respecting the efficacy of melody in numerous cases of insanity. On this important point, the authority of Scripture, as also of ancient and eminent modern authorities, is highly favourable to employing music towards alleviating paroxysms of mania; indeed, it may be asserted confidently, that often efficacious remedy is now too much neglected.

After work or amusement, parties of lunatics—both male and female—frequently go out towards evening to walk in the neighbourhood, or upon the boulevards of Auxerre: which, for beauty and splendid prospects they nearly everywhere afford, are unique, if not the most charming promenades throughout France. On no account whatever, are patients ever permitted to enter the town, when enjoying these excursions, lest the proceeding might prove inconvenient to the inhabitants, besides acting injuriously upon inmates. This is an excellent regulation, and highly commendable. During the afternoon of my visit to

Auxerre, two parties of maniacs, consisting each of about fifteen females, with attendants, left the asylum for an excursion in the adjoining fields. Having previously arranged their dress, put on gay caps and shawls, the groups really looked more like countrywomen going to a fair or merry meeting, than lunatics leaving a mad-house. The spectacle thus brought accidentally under observation was exceedingly gratifying; particularly, as the persons then setting off to breathe cool breezes in the open country around seemed highly pleased, and behaved as if rational beings, thereby justifying a proceeding so deserving of general imitation.

Besides occupying inmates in physical labour, according to each individual's capabilities, mental culture is likewise carried forward assiduously. The chief means then employed are reading, writing, arithmetic, and drawing, whenever suitable to each pupil's varied capacity: the system pursued being by mutual instruction. During the period patients were engaged in these exercises, I visited various parties, both in the male and female departments, where it was exceedingly interesting to see insane teachers instructing other lunatics, from the first elements of education up to drawing, and to repeating from memory passages of authors. Several male inmates wrote admirably, although some could scarcely hold a pen when first admitted; and many, who could neither read nor write on entrance, had since become proficients in these accomplishments. Notwithstanding particular patients acted as monitors, still professional instructors—both male and female—were attached to different divisions, who taught the monitors and more advanced inmates, besides superintending the whole proceedings: which, I can state, have already produced very beneficial consequences.

In some of the new work-rooms recently constructed—all of which are lofty, well ventilated, and cheerful looking—various appropriate mottoes, and instructive sentences or proverbs, have been written in large characters upon the sides of each apartment. Being constantly under the observation of residents, besides attracting their attention, and so exciting mental volition when read, they also convey instruction. To copy in my present narrative any of the adages thus recorded is unnecessary, seeing many were appropriate sayings and quotations from holy writ. The plan itself, however, cannot be too much lauded: and deserves being brought under notice, for the information of other official authorities. Like sermons in stones, and good in every thing, to find madhouse walls thus teaching wisdom to human beings deprived of reason, is certainly gratifying evidence of the great superiority of this institution. Flowers, with other ornaments, were likewise placed on the tables and mantel-pieces; whilst scrupulous cleanliness prevailed everywhere.

In the dormitories, all the bedsteads were of iron, and those occupied by dirty patients seemed of a novel yet excellent description. In the various divisions an accurate register is kept, as well of the description of labour, as of the amount performed by every patient, with the number of hours each individual was employed; the value of the work accomplished, and other particulars, were likewise all accurately recorded. At the end of every three months, the whole particulars are then regularly entered against each patient's name; so that the committee of surveillance can at once ascertain how different inmates have been engaged, and the gratuities to which any person is entitled.

Another arrangement, equally important in reference to the lunatic's well-being and comfort, deserves even more than a passing remark, since it produces exceedingly beneficial effects throughout the establishment. The measure alluded to has now been in operation during some time, and confers much credit upon Dr. Girard for its introduction. According to existing regulations, each patient in the asylum must have a "trousseau," or "kit," to use military language, which comprises three changes of linen, such as shirts, stockings, and handkerchiefs, besides a comb and brush. Upon every separate article a specific number is marked—originally given to the party on entry—but never assigned to any other inmate: or altered, however long its possessor remains in the establishment. The system invariably pursued respecting trousseaux is the following:—One change is worn, another is at the washhouse, and the third lies ready for use on a particular shelf in the dormitory, on which each owner's number is inscribed; while the brush and comb are always suspended from every patient's bed-post, when no longer required. Besides preventing any inmate from wearing another's clothes, which becomes both useful and essential, especially where females are congregated, this arrangement also obliges parties to arrange separately their own habiliments. But other advantages of even greater importance deserve notice: since such means promote cleanliness, and also awaken order, with a desire to preserve individual property. Judiciously managed, the plan often produces beneficial consequences; it teaches also regular habits amongst residents, and even induces mental application. Dr. Girard spoke highly of the important results observed by lunatics being thus obliged to superintend their own trousseau; and he strongly recommended its adoption in every lunatic institution.

Much attention is likewise paid to the proper classification of patients in this asylum; although during the demolition of ancient dormitories, and the construction of new buildings, this cannot be properly accomplished, especially owing to deficient accommodation, on the male side, where various sleeping apartments and work-rooms are over-crowded. Notwithstanding this inconvenience, and the large number of ordinary workmen now occupied on the premises, great quietude prevailed everywhere; indeed, no confusion or apparent disturbance seemed produced by lunatic labourers mixing with, and assisting others perfectly sane. In proof of this it may be mentioned, when the bell rang after dinner for workmen to resume labour, a number of patients took up hods or shovels, apparently with as much alacrity and calmness, in order to join other labourers, as if no distinction whatever existed respecting their mental faculties.

Subsequently, I visited, with Dr. Girard, several refectories, when residents were partaking their evening meal. In one apartment, ninety patients sat at table, and behaved like rational creatures. Each had knives, forks, and napkins; whilst flowers in pots enlivened the scene, and served as ornaments, alongside the dishes containing viands. Each group, or mess, was presided over by a patient belonging to their own party; and although ordinary attendants were present, several lunatics likewise acted as assistants, being distinguished from ordinary inmates by wearing a particular coloured jacket, to give them official authority. Afterwards, all the guests returned to their respective court-yards, to repose in the shade, or to breathe a little fresh air, previous to resuming work, amusement, or instruction.

From the ample opportunities afforded of seeing the entire population, I am enabled to say confidently, the physical health of the whole establishment was satisfactory. Indeed, scarcely any bodily sickness prevailed, as few inmates occupied the infirmary, and those actually in this department only suffered from insignificant complaints. Whether labouring under mental maladies, or suffering from any bodily disease, accurate reports of every case are regularly taken by the physician or interne, which contain very full particulars of the history, symptoms, and treatment pursued. I specially mention this circumstance, as the official case-book seemed more full of useful details, than similar documents in many other insane institutions. The system adopted when compiling these records, to my mind, seemed concise, yet minute, and exceedingly instructive.

Regarding the medical treatment of mania, I would here remark that, Dr. Girard entertains a most favourable opinion of sulphate of strychnine, as an efficacious remedy in cases of idiocy, stupidity, and general paralysis of the insane; more especially, where the individual is a dirty patient, of which several examples, illustrating the utility of this preparation, were pointed out during my perambulations. When exhibited, the beneficial operation of strychnine often appeared most marked; as patients were then enabled to use their ordinary clothing, and to occupy a common apartment, which they seldom dirtied if under its influence. The medicine frequently effected so much good in cases of this description that, the average number of dirty patients has been reduced to four, or at most to five, throughout the asylum. Besides these beneficial results, lunatics taking strychnine often become less apathetic, and are also more easily induced to work at any employment than previously. Dr. Girard further mentioned, he had observed decided curative effects to follow the administration of ergot of rye, if given in large doses to female patients labouring under intermittent mania, especially if the attack depended upon, or was influenced by, disordered catamenia. Several instructive examples of the benefits thus derived were shown subsequently. However, as that physician intends soon to publish his own remarks, and recent experience in regard to the above remedy in intermittent mania, the profession will then be able to judge for themselves respecting its utility.

Unlike many public lunatic establishments in France, the Auxerre Asylum is amply supplied with excellent water, recently brought at an expense of 1300*l.* from St. Margaret's spring—situated on a neighbouring elevation. Further, as a large reservoir has been constructed within the premises, water is now laid on to every court-yard; but although it has not yet been carried into the different buildings, the supply is most plentiful, and proves a great acquisition to the entire establishment. A new bath-house also enables the executive to administer bathing in every form; which here, as elsewhere throughout the country, is much employed as a remedial measure during the treatment of insanity.

Although the present garden adjoining is not of limited extent, it will soon be considerably enlarged by a new purchase lately made: whereby, the ground appropriated to horticultural occupations will amount to about twenty-five acres. No farm is attached to the asylum, and there does not appear to be any intention of acquiring such an appendage; as it is thought more advisable to employ lunatics in garden work, than the former more laborious occupations, which cultivating a farm necessarily requires. Dr. Girard, however, highly approves of gardening employments, and thinks that kind of bodily labour not only produces sufficient muscular exertion, but is, besides, much more agreeable to lunatics, than almost any other kind of out-door work. At the same time, the culture of esculent plants, and the pleasing aspect of flowers, generally produce favourable impressions upon the obtuse or weakened intellects of lunatics, and may more often prove efficacious.

Cholera prevailed, during 1849 to some extent in this asylum, when twenty-one deaths occurred from that epidemic. Again, as the facts may seem interesting, I should likewise mention, in reference to the total movement of patients that, during ten years ending on the 31st of December, 1850, as many as 878 insane patients have been admitted, of whom 236, or nearly 26 per cent., were discharged cured; seventy-three left the institution improved; eight also left for various reasons; and 286, or 32·57 per cent. died; thus leaving 275 lunatics under treatment on the 1st of last January. It ought also to be added, amongst the entire number, 246 were pensioners, and 632 indigent patients; whilst 272 of the latter category were tranquil, and 360 classed as dangerous. It is likewise instructive to state, seeing the facts partly explain the small amount of cures, and large ratio of deaths that, nearly all the 272 inmates reported as inoffensive patients, were old people, who laboured either under senile and simple dementia, general paralysis, or chronic epilepsy without delirium, many being besides idiots and imbeciles.

When all the improvements at present in progress, and those contemplated, shall be completed, unquestionably the Auxerre Asylum will then become one of the best constructed throughout the French republic.

Already from six to seven hundred thousand francs have been expended in rearing the new constructions; and as an additional two hundred and fifty thousand francs were voted by the departmental council-general, only the week previous to my inspection, in order to complete this institution, nearly one million will ultimately have been spent upon the entire structure. Much of its actual efficiency, and acknowledged reputation amongst the public asylums of France, is certainly due to Dr. Girard's unwearied professional exertions, and directorial superintendence. But, however true the above remarks, it is equitable also to state that, other parties likewise deserve much credit in the same benevolent undertaking; and no one more justly than the present Préfet, M. Haussman, who now fills that high government appointment. This gentleman has all along taken the greatest interest in the progress and improvement of the Auxerre asylum. Indeed, it has been a fortunate circumstance that a person so influential in the department as he is virtually, seems a zealous and energetic promoter of all judicious ameliorations. Through the influence of such an authority—the ruling executive power in his own district—former and recent grants of money have been more easily obtained, and difficulties were thereby removed, which might have proved otherwise insurmountable. Having had the honour of an interview with the Préfet, who received me at the préfecture in a most urbane manner, I would briefly observe, before concluding the present narrative that, amongst numerous interesting remarks then made to Dr. Girard and myself, it was exceedingly satisfactory to hear many practically sound views so clearly developed at head-quarters, respecting the judicious management of lunatic asylums. Entertaining highly-pleasing reminiscences of this conversation, besides other satisfactory circumstances, I left Auxerre sincerely desirous every French departmental institution might always obtain official rulers equally able and zealous like M. Haussman, who was, to adopt the English designation, Lord-Lieutenant of the Yonne.

### DIJON ASYLUM.

The above-named institution for lunatics is situated within a very short distance of Dijon, formerly the ancient capital of the powerful Dukes of Burgundy, but now chief town of the Côte d'Or department, and containing about 26,000 inhabitants. The ground occupied by this asylum once belonged to a Carthusian monastery, whereof a few remains still exist, especially the celebrated Well of Moses, executed by Claus Slater. This curious structure contains statues of Moses, Daniel, David, Jeremiah, Zechariah, and Isaiah, each placed upon elaborately chiselled pedestals; and as every figure seems admirable in expression, they deserve examination. The well in question occupies one of the asylum court-yards; where many strangers come to admire so singular a work of art, from being considered one of the most interesting relics of olden times, throughout this part of France. Some remains of an old chapel also exist, which constituted the ancient burying-ground of the reigning ducal family. Amongst the most remarkable tombs this cemetery contained, that of "Philip the Bold," and also of his son "John Without Fear," stood pre-eminent: both being reputed the finest specimens of medieval art existing north of the Alps. The last-named duke, our readers may remember, was most barbarously murdered upon the Bridge of Montereau, on the 10th of September, 1419, during a conference with the Dauphin, afterwards Charles the Seventh of France.

Notwithstanding the iconoclastic fervour of the revolutionary commune, who had decreed, in 1793, these monuments to destruction: nearly every portion of both tombs was fortunately preserved, either in private cabinets, lumber-rooms, or the church of St. Bénigne, till 1818, when, the departmental authorities having determined to restore these sepulchral antiquities, the different pieces were carefully collected, repaired, and ultimately placed in the public museum; where they will amply repay inspection by artists, or amateurs of beautiful and elaborate workmanship.

The Dijon asylum is situated close to the city, and not far from the Paris railway station. Notwithstanding its vicinity, the building is not overlooked even by immediate neighbours, in consequence of a row of trees which interrupts their view, whilst a thick wood occupies the opposite quarter. Further, the gardens and fields belonging to this institution being surrounded by a high wall, not only all exterior communication is prevented, but strangers can neither see the interior, nor any inmate observe or be disturbed by outward objects. Although the asylum is constructed on rather low ground, and has moderately elevated hills adjacent, nevertheless, the locality is considered salubrious. Many ancient French monasteries having become ruinous through age, or been demolished during the first revolution, scarcely any part of the old buildings now remain, excepting the director's residence, and a wing now appropriated for offices. The dormitories, and court-yards, with other appurtenances, are all new constructions, specially erected for the reception of lunatics, who have been admitted, since 1843, into this public asylum; of which the medical staff comprises a physician-director—Dr. Dumesnil, and one interne, both being resident.

At the period of my visit, the number of insane patients in the Dijon asylum amounted to 254, of whom 101 were males, and 153 females; about nine-tenths of the whole being considered incurable. With reference to the general classification of inmates under treatment, it will sufficiently indicate several chief features to mention that, eleven laboured under paralysis, comprising eight males, and three females; forty-one were epileptics, twenty being men, and twenty-one women; whilst about one-seventh of the total number, or thirty-six persons, were classed as dirty patients. Although indigent lunatics belonging to the Côte d'Or can only be admitted into this public asylum, private persons are received, as at other departmental institutions for the insane throughout France; the payment ranging, in such cases, from 500 to 3000 francs per annum, according to the accommodation supplied. At the period previously quoted, the number of pensioners amounted to thirty-seven, of whom fifteen were male and twenty-two female maniacs.

Respecting the important question of personal coercion, it is satisfactory to report, not one male patient was restrained by a strait-waistcoat, on the morning of my inspection. Five female inmates were, however, then in camisole; one of whom had the face likewise covered with an iron-wire mask, to prevent her tearing the clothes she wore, being often much excited. Nevertheless, it should be stated, in one of the court-yards occupied by males, an idiot patient was loosely attached to the iron railings of the enclosure, by a broad belt, to prevent his falling down, whilst inhaling fresh morning breezes; but, otherwise, he continued quite free. The above precaution had been only adopted, because this poor fellow could scarcely stand upright, and was frequently violent, besides being dangerous to other inmates. Indeed, some months before, having attempted to walk in the court-yard, he fell and broke his arm, which had only recently united. After such an accident, it was thought most prudent, whenever this patient remained out of doors, to adopt the loose strap just mentioned, so that no other mishap might again supervene.

The general health of residents appeared uniformly good, very few patients being then in the infirmary suffering from bodily disease: and even those actually under medical treatment, in this division, were only affected with unimportant complaints. Indeed, the salubrity of the asylum, and physical condition of its entire population, were reported to have proved equally satisfactory during the past three months, not one patient having been confined to bed continuously throughout the establishment. When perambulating various court-yards, two goitreuse females were noticed; still this affection is not considered common in the surrounding district. Epileptic and paralytic patients, as already remarked, were rather numerous. Respecting the latter malady, I would here mention an observation which Dr. Dumesnil made, as it seemed important, viz., paralysis would not so frequently afflict insane people resident in asylums as at present, were they always to have plenty of fresh and well-cooked vegetables, along with their usual provisions. In fact, he viewed this disease in a light somewhat similar to the scurvy, which often attacks persons deprived for a long time of these essential articles of diet. This opinion being based upon considerable experience, obtained in other establishments, and as the observation is supported by such good authority, it deserves notice in these pages, so that other practitioners may make further inquiry regarding similar practical questions.

Although cholera prevailed epidemically in Dijon, during 1849, no case of that disease actually occurred in the asylum. However, respecting the general mortality met with, it will be interesting to add that, throughout the three last years, viz., 1848, 1849, and 1850, the average rate has amounted to one fatal case in about every fourteen and a half inmates; thus, making seven deaths per hundred admissions. Amongst these examples, a large majority were occasioned, according to subsequent autopsies, by diseases of the head and nervous system, three-fifths, at least, having been of that description; whereas, very few exhibited any affection of the thoracic organs, not only then, but ever since the asylum was first opened.

Such observations become highly instructive; especially as they are derived from minute examination of the register of dissections made at the Dijon asylum, since 1843, which Dr. Dumesnil kindly permitted me to inspect. No evidence could be more conclusive regarding the infrequency of pulmonary complaints amongst lunatics, throughout this part of France, than the numerous necrotomies thus performed. The above fact is farther of great practical importance, seeing it clearly indicates this locality, instead of proving prejudicial in pectoral diseases, or likely to produce them, has an opposite tendency; since the maladies usually very fatal amongst insane patients are pulmonary, which seem here exceedingly rare. Being unable to make inquiries at the city general hospital, in order to ascertain whether this peculiarity also prevailed in ordinary patients, it is, consequently, impossible now to draw general conclusions respecting the prevalence or rarity of pectoral complaints in this district. Nevertheless, the point now mooted deserves further investigation: more especially, as the experience acquired at the Dijon asylum, during several years, would certainly warrant an opinion favourable to the Côte d'Or climate, in reference to pulmonary disease, compared with some other departments. Having purposely alluded to the above unusual feature exhibited at this institution, not only on account of its scientific application, but also to induce other physicians to make additional inquiry, I shall feel satisfied, should the few cursory remarks now made have such results; because, if it be afterwards shown that phthisis seldom prevails in this locality, then medical knowledge will become extended with benefit to humanity.

During 1850, the movement of insane patients at the Dijon asylum, according to the official register, was reported as follows:—

| | Males. | Females. | Total. |
|---|---|---|---|
| Admitted . . . . . . . . | 42 | 50 | 92 |
| Discharged Cured . . . | 15 | 13 | 28 |
| Died . . . . . . . . | 6 | 14 | 20 |

From these data it appears, not only fewer female patients left the asylum convalescent, during last year, but the amount of deaths, amongst that class, likewise predominated considerably. This peculiarity in the above report becomes more remarkable, seeing the result is somewhat different from observations made elsewhere. However, the statements now recorded, may be perhaps exceptional, as Dr. Dumesnil's experience, during the present year, indicates that the proportion of patients discharged cured, will be larger than in the previous season. This inference he based upon the circumstance of more recent cases of insanity having been received, during the present, than the past year, when the aggregate number of epileptic and incurable lunatics admitted were much more numerous. In corroboration of such remarks, and as otherwise interesting, it was further stated that, a male patient had been recently discharged cured, after suffering, almost every ten days, severe attacks of epilepsy. Besides this fact, two epileptic men ought likewise to be mentioned, who had remained totally free from that terrible malady; one during three, the other about two years consecutively.

The various court-yards of this asylum are open, airy, well ventilated, cheerful, and spacious. The dormitories, like many others recently built throughout France, seemed of a superior description; and windows being on each side, they had by no means a sombre aspect. The bedsteads were all of iron; but being rather numerous, in various sleeping apartments, the patients there appeared too much crowded.

Being constructed upon sloping ground, several divisions of the asylum hence became elevated above the portion immediately adjoining; whereby, the second story of one building seemed, in some places, almost on a level with the basement of its adjoining dormitory. Considering inmates of one court-yard could easily observe residents in the neighbouring enclosure, this feature was highly objectionable, and detracted from arrangements which merited otherwise much commendation. Further, as one structure, when more elevated than another adjoining, may engender damp in lower portions, during rainy weather, similar to the Clermont asylum previously described, every institution containing numerous patients should therefore be always placed upon a nearly level foundation. Again, in each division of the asylum, open galleries have been constructed, where lunatics can take shelter against rain or sunshine, besides being thus enabled to pursue, in the open air, various occupations. These essential appendages are exceedingly convenient, since, however unfavourable the weather may occasionally prove, no inmate need ever be confined to close apartments during day-time, nor breathe a vitiated atmosphere.

Speaking generally, this establishment exhibited an aspect of tranquillity; the women being certainly rather quiet, although not so much as occasionally noticed elsewhere. Occupying insane patients, in some manual and varied employment, appeared a great object with the authorities; since, the more lunatics are allowed freedom, they will less likely become excited, violent, or dangerous. About half the entire number of inmates are usually occupied: the men at various employments, some in the gardens, in trades, or handicrafts, and so forth. The women were sewing, knitting, making and mending clothes, or busy in ordinary household occupations. Even amongst the agitated patients, I observed several females knitting stockings assiduously, notwithstanding their violent conversation and excited gestures. Hence, endeavouring to occupy the mind diseased through bodily labour, seemed constantly kept in remembrance.

No resident ever leaves this asylum to labour elsewhere; and as all are exclusively engaged on the premises, with the exterior they hold no communication. Being without any farm attached to the institution, those patients who are occupied out of doors, labour in the extensive gardens adjoining, which contain about twenty-seven acres. When perambulating the grounds, I noticed two or three parties of inmates busily employed; some in raising potatoes, others were making a new gravel walk, and several in ordinary horticultural occupations; both male and female patients being thus engaged.

Within the inclosure, a small river having been converted into a kind of pond, an excellent place for open-air bathing is thus obtained. Here, sometimes, forty male patients enjoy a cool dip, in the pure stream; whilst some even amuse themselves with swimming. Dr. Dumesnil approves highly of such an amusement amongst lunatics, and therefore he encourages its adoption during fine weather; of course, taking care to have sufficient attendants always present. Indeed, this recreation is so much appreciated by many inmates that, to be allowed to bathe in the river, is often considered a special favour granted by the physician; whereas, being debarred is looked upon as a punishment. Besides conducing to cleanliness, cold bathing thus employed improves the physical health of various patients, and also seems to alleviate their mental malady. The female lunatics likewise possess an appropriate locality at another part of the river, where they bathe; and it was said, even swim occasionally. Being the only instance of river bathing, in the open air, permitted to insane patients, which has come within my immediate observation, I have consequently felt more desirous of noticing this system adopted at the Dijon asylum; believing it worthy of imitation, on Dr. Dumesnil's authority, who spoke favourably respecting the remedy, after considerable experience.

In addition to such an ample source of water, for open-air bathing, this most essential element is otherwise abundantly distributed to the various buildings; a further supply having been recently brought, at an expense of 26,000 francs, from high ground in the neighbourhood. Already, capacious pipes are carried up to every floor; and a large cistern being placed at the top of each flight of stairs, near the dormitory entrance, water for every purpose remains constantly accessible. Consequently, in the above respect, compared with other asylums throughout France, the Dijon certainly stands pre-eminent. Water being now so plentiful, one of the proposed ameliorations, which will be soon commenced, is a new bath-house; since the present building has become altogether inadequate; more particularly, while bathing is held by French physicians in high estimation as an efficacious remedial agent, during the treatment of insanity.

Other improvements are likewise in contemplation; and so soon as these are completed, the classification of patients will be further extended. Compared with more ancient establishments, the dormitories of the Dijon asylum are infinitely superior; nevertheless, in some respects, the new apartments and cells for secluding excited patients appeared not so good as those of a similar description at Châlons. However, the entire structure is otherwise excellent; and having windows on each side, the apartments seemed cheerful, well ventilated, and salubrious. In addition to these important requisites, the dormitories being all without iron bars, whilst they had no prison-like appearance, although light wire trellises were placed outside, to prevent accidents, the accommodation was altogether unexceptionable.

In consequence of the present residence for private patients being of limited extent, and as the authorities consider it very desirable to improve the style of these apartments, new buildings and flower gardens for that class of inmates are proposed; which, doubtless, will be provided. An official residence for the physician is also projected, when the last remaining portion of the monks' former dwelling will be entirely demolished. Another intended alteration ought not to be forgotten, seeing the change proposed must prove, for various reasons, both judicious and beneficial. I here allude to filling up a small lake or pond, in the adjacent lawn, where the holy fathers formerly preserved fish alive, and fattened them previous to cooking. The monks being ichthyophagists, according to common report, it consequently became a matter of much importance to all true Carthusians, if they could procure plenty of the finny tribe—savoury and fresh, for daily consumption; since fish constituted their staple article of food. Hence, the lake or "piscina," in question. But times, as also many ancient customs, are now altogether changed; and this stagnant pool is no longer required for its original purpose. Nay, as such places engender damp, and often produce malaria, besides being dangerous appendages to a madhouse, the ground should be immediately converted into flower parterres, or covered with greensward; whereby, it would become an additional locality for recreation.

Retaining various agreeable reminiscences of my visit to the Côte d'Or asylum, and especially acknowledging the kindness I experienced from Dr. Dumesnil, during the many hours passed in his company, it ought to be added, in justice to the departmental authorities that, the institution they have recently erected, for affording relief to a most unfortunate class of their fellow creatures, is highly creditable, and has proved of great advantage to suffering humanity. There, as elsewhere, laudable efforts are constantly made to alleviate the afflictions of numerous persons who, in ages not very remote, were treated more like animals, than human beings, although endowed with life and all its wonderful attributes, however deficient they appeared in their mental faculties. At present, matters are greatly changed; and in the race of improvement prevailing throughout most parts of civilized Europe, towards ameliorating the lunatic's suffering condition, France has attained a highly prominent position, which other countries still lagging behind, in many important regards, would do well to imitate.

So much for the Dijon lunatic institution; about which I could easily add other observations: but refrain, lest the previous narrative should appear already sufficiently extended. However, before taking leave of the ancient capital of Burgundy—so remarkable for many interesting associations, beautiful promenades, and various ancient relics still extant, illustrating especially, for so early a period, really advanced civilization—I would allude to one circumstance that came under my observation as a traveller, which, although its effects may not act directly upon rational and responsible human beings, nevertheless still deserves incidentally a passing notice. The point referred to is therefore now detailed, by way of digression, notwithstanding it may appear to readers somewhat irrelevant, even while considerable physical suffering, if not disease, thereby supervened.

Feeling desirous of inspecting every interesting object deserving notice, in the former Burgundian capital, amongst the places I accidentally visited, when roaming about in search of old churches, and other remains of antiquity, chance led me to the cattle-market, near an old city gate. Being very soon after sunrise, the place was crowded, by both country and towns people; whilst much business appeared then transacting, relative to various objects, which seemed, at first sight, the carcases of lambs and calves. These lay on straw, in rows, and were arranged much in a similar manner as dead geese and chickens are placed in the shops of London poulterers. At first, I concluded this locality was for selling dead, not animated creatures. However, to my great surprise, on examining several animals, then stretched on the cold ground, motionless, and scarcely appearing to breathe, they were actually living calves, benumbed, and almost in a state of asphyxia. The four feet of all were firmly tied together by strong ligatures, whilst a rope was also around the neck of each; whereby, these helpless sufferers could neither move, nor change position. Indeed, they really appeared more dead than alive, in consequence of having remained in this tortured condition, during many hours, subsequent to leaving their native village folds. Upwards of a hundred tormented calves and lambs were subjected to this unfeeling treatment, which is universally practised at Dijon; and a bystander then admitted, in answer to my inquiries, that three, or even four hundred young calves were sometimes similarly tortured. When any bargain was concluded, having first made a mark with scissors, which also drew blood in some instances, the butcher now grasped the cord attached to the animal's neck, whilst an assistant took hold of its feet with one hand, and wound the pliant tail round his other; whereupon they tossed their dumb victim, by an united effort, into a cart, in which it was carried off for sacrifice at the city *abattoir*.

The atrocities often ascribed to inferior Smithfield functionaries are certainly bad enough, and should be suppressed; but I much question, if even in that blood-stained spot, any sentient beings were ever systematically treated, after the manner now described as common in the Dijon calf-market, and at other places according to report. Such cruelties must be abated; seeing, the treatment thus inflicted upon living creatures, endowed with acute physical sensations, is unjustifiable, besides being highly injurious to meat intended for the food of man. On that account, irrespective of much higher motives, the barbarous custom here condemned ought entirely to cease, wheresoever it may actually prevail.

### MARÉVILLE ASYLUM.

This extensive public asylum was originally founded as a pest-house, in 1597, by a young lady named Anne Feriet; for which specific purpose, the institution continued appropriated during forty years afterwards. Subsequently, it became a mendicity depôt, when not only lunatics, but *mauvais sujets* belonging to the district, were admitted. The place also served as a Bastile for the confinement of state prisoners, sent by *lettres de cachet;* whereby, these victims of tyranny were sometimes shut up for life, and often remained completely forgotten. From 1749 to 1794, the establishment was administered by a religious society denominated "*Les Frères de la doctrine chrétienne.*" At the revolution, Government, however, materially altered its internal management, especially after 1794, when only insane patients were received; for which purpose it has been ever since appropriated. At present, the asylum admits indigent lunatics from five neighbouring departments,—namely, the Meurthe, Vosges, Moselle, Haute Saone, and Ardennes; besides which, the department of the Seine has always a hundred beds at the disposal of Parisian authorities. However, pensioners are received from any district, and some even belong to foreign countries.

Maréville is situated on the eastern declivity of a rising ground, which forms a kind of amphitheatre open at one side, and nearly a league distant from Nancy, the ancient capital of Lorraine, but now chief town of the Meurthe department. Nancy contains 36,000 inhabitants, and is truly one of the prettiest provincial cities in all France, on account of the width and regularity of its streets, besides various beautiful public buildings, whereby, it deserves this well-merited distinction. The palaces, numerous triumphal arches, and other interesting objects ornamenting this city, ought certainly to be inspected by all travellers, whether professional, or mere seekers after novelty. Amongst the different *notabilia* deserving inspection, the most remarkable were either constructed, or greatly embellished by Stanislas de Lescynski, ex-king of Poland, who had retired thither, after abdicating the elective Polish crown, although he still retained his hereditary territories and title, as Duke of Lorraine and Bar. This eminent patron of arts, sciences, and literature died in 1766, having been accidentally burnt to death by his clothes taking fire, whilst quietly sitting in an apartment of the palace. To commemorate the many benefits conferred on his native country, by this accomplished prince and ex-sovereign, a magnificent monument was subsequently erected in the modern Place du Peuple, but *ci-devant* "Royale!"

The road leading from Nancy towards Maréville was interesting, as it passed over the battle-field in which Charles, "the Bold," Duke of Burgundy, sustained a complete defeat in 1477, when besieging the city, and where he afterwards lost his life by being drowned in an adjoining marsh. Before approaching the asylum, its numerous buildings seemed picturesque objects in the distance; and on drawing near, various anticipations already figured themselves in my imagination, which were fully confirmed by subsequent personal observation.

The medical staff of this asylum consists of a chief physician—Dr. Morel de Gany, most favourably known, not only in France but throughout Europe, by his various publications; also an assistant-physician—Dr. De Roche, and three internes, with one pharmacien. To these resident officers the present director may be justly added, seeing he is a gentleman of celebrity and great reputation, namely—Dr. Renaudin, who was formerly chief medical superintendent of another public lunatic asylum, besides being the author of various valuable works upon mental diseases and the management of insane institutions.

On the day of my visit to Maréville, the total insane residents amounted to 876, of whom 471 were males, and 405 females; amongst these, however, 117 were classed as pensioners, 77 being male, and 40 female patients. The sum charged for such cases varied from 400 to 1000 francs per annum, with 600 francs additional, when a special attendant was required for the service of one individual. At least four-fifths of the resident lunatics were incurable, some having been even forty years insane. According to the registers, 34 inmates, or 28 men and 6 women, laboured under general paralysis; 60 were epileptics, 32 being males, and 28 females; whilst the entire population only contained about 50 dirty patients. Completely at variance with the statistical statements obtained at several asylums previously inspected, the figures now recorded show that male lunatics under treatment in Maréville exceeded the proportion of females by 66, thus giving an excess of 13·58 per hundred in the former sex. The above fact is important, as it apparently indicates greater liability to insanity amongst the male than female population; which conclusion becomes more instructive, seeing it applies, not to any particular district, but to the five departments from whence indigent insane patients are usually sent to this public establishment. Reasoning, likewise, from other data equally authentic, it may be further stated that, mental diseases occur much more frequently throughout urban than rural portions of the community, not only in Lorraine, but its adjoining provinces; the proportion being nearly double the former, compared with the latter class, especially in the Meurthe department, where, it is reported about one mad person is met with, in country districts, for every 1468 inhabitants: whereas in Nancy, the ratio actually reaches to one lunatic for every 500 inhabitants.

During 1850, the following official figures indicate the movement of patients at this public asylum :—

| | Males. | Females. | Total. |
|---|---|---|---|
| Admitted | 108 | 90 | 198 |
| Discharged cured | 24 | 17 | 41 |
| Died | 44 | 40 | 84 |

Besides these numbers, it should be added, that nineteen patients escaped during the year, of whom fourteen were subsequently brought back to Maréville; and further, amongst the deaths enumerated, two suicides are included, one being a male, and the other a female patient.

In order to illustrate several characteristic yet prominent features manifested by various cases enumerated in the preceding statement, I have constructed, from authentic returns—obligingly supplied by MM. Renaudin and Morel, the subjoined tabular analysis of the mental disease which affected lunatics admitted, also the number discharged convalescent, and lastly, the deaths recorded, during twelve months. From these details it will be readily perceived that, in numerous patients placed under treatment, slight hopes could be reasonably entertained of permanently ameliorating their mental malady, whilst recovery was nearly impossible.

*Synopsis of the Diseases, Admissions, Cures, and Deaths, recorded amongst Insane Patients, at Maréville, during* 1850.

| TYPE OF DISEASE. | ADMITTED. | | | CURED. | | | DIED. | | |
|---|---|---|---|---|---|---|---|---|---|
| | M. | F. | Total. | M. | F. | Total. | M. | F. | Total. |
| Acute Mania . . . . | 31 | 23 | 54 | 18 | 10 | 28 | 10 | 5 | 15 |
| Chronic Mania . . . | 9 | 4 | 13 | 2 | — | 2 | 4 | 3 | 7 |
| Intermittent Mania . . | 5 | 2 | 7 | — | — | — | — | 1 | 1 |
| Moral Mania . . . . | 5 | 1 | 6 | — | — | — | — | — | — |
| Lypemania . . . . | 8 | 20 | 28 | 2 | 7 | 9 | 1 | 2 | 3 |
| Monomania . . . . | 5 | — | 5 | 2 | — | 2 | — | — | — |
| Hypochondria . . . | 3 | 1 | 4 | — | — | — | — | — | — |
| Dementia . . . . . | 6 | 8 | 14 | — | — | — | 6 | 14 | 20 |
| Paralysis . . . . . | 11 | 7 | 18 | — | — | — | 8 | 7 | 15 |
| Epilepsy . . . . . | 10 | 4 | 14 | — | — | — | 9 | 4 | 13 |
| Idiots and Imbeciles . | 15 | 20 | 35 | — | — | — | 5 | 3 | 8 |
| Suicides . . . . . | — | — | — | — | — | — | 1 | 1 | 2 |
| Totals . . . | 108 | 90 | 198 | 24 | 17 | 41 | 44 | 40 | 84 |

Restricting my present remarks to the four subdivisions which embrace dementia, paralysis, epilepsy, and idiots with imbeciles, it will be observed that, eighty-one persons, or nearly forty-one per cent. of the whole number admitted, were afflicted with the above almost incurable types of insanity. Further, no patients classed under any of these denominations left the asylum convalescent, whilst fifty-six deaths, or 66·66 per cent. of the eighty-four fatal cases reported, were of that description. Again, respecting the amount of cures and deaths recorded in other varieties of mental disease, it will be also perceived, although the largest proportion of recoveries occurred in persons affected with acute mania, the ratio of mortality also proved considerable in that division; both results being more numerous amongst male patients. The most fatal malady was, however, dementia, by which inveterate disease twenty deaths supervened, and by paralysis fifteen; thus showing that, thirty-five fatal cases, or 41·66 per cent. of the mortality, from all causes, was produced by these two incurable forms of insanity. Typhoid fever having attacked various epileptic male patients, in consequence of the dormitory they then occupied being in an unhealthy condition, that circumstance tended to augment the number of fatal cases amongst these afflicted inmates. This part of the building being now demolished, the alterations will doubtless be productive of improved salubrity henceforth in that division.

As many insane residents at Maréville belong to the middle classes of society, it will be interesting to state that, thirty-nine new patients of the above description were admitted during last year; eleven were discharged cured, eight left the institution prior to the complete restoration of their mental health, whilst nineteen died. This constitutes a large proportion, and in some respects seems a high rate of mortality, considering the number of pensioners usually resident; from whom 56,837 francs were received during last year for their support and medical treatment in the establishment.

Another feature also of importance, regarding the patients admitted during 1850, should not be overlooked, namely, the particular season when mental diseases most frequently prevailed throughout the various districts of this part of France. Such an inquiry is instructive, seeing several practical deductions may be based upon correct statements. For example, during the first six months of last year, eighty-seven lunatics were admitted into Maréville; whereas, in the remaining two quarters they amounted to 111 patients. From these data it may be fairly inferred that, insanity is more frequently developed during warm weather than at any other season: which result entirely coincides with conclusions elsewhere deduced, besides being supported by general experience.

Maréville being one of the largest public lunatic asylums in France, any analysis illustrating the various forms of mental disease affecting its numerous population therefore becomes exceedingly interesting: not only on account of many important details thus brought forward, but also in consequence of such statements enabling investigators to arrive at correct notions, respecting the most common varieties of mental disease prevalent in particular provinces. With that object, the subjoined table has been compiled from official documents, to which I would now direct attention before deducing any conclusions.

*Form of Disease affecting the* 809 *Insane Patients resident at Maréville, in January,* 1851.

| DISEASE. | M. | F. | Total. | DISEASE. | M. | F. | Total. |
|---|---|---|---|---|---|---|---|
| Acute Mania . . . | 22 | 20 | 42 | Brought up . . . | 289 | 276 | 565 |
| Chronic Mania . . | 75 | 96 | 171 | Epilepsy . . . . | 35 | 21 | 56 |
| Intermittent Mania . | 15 | 8 | 23 | Paralysis . . . . | 14 | 6 | 20 |
| Moral Mania . . . | 13 | 6 | 19 | Idiots . . . . . | 28 | 10 | 38 |
| Lypemania . . . | 29 | 41 | 70 | Imbeciles . . . . | 44 | 48 | 92 |
| Monomania . , . | 20 | 4 | 24 | Weakminded . . . | 7 | 7 | 14 |
| Hypochondria . . | 5 | 1 | 6 | Simpleminded . . | 13 | 7 | 20 |
| Dementia . . . . | 110 | 100 | 210 | Crétins . . . . . | 2 | 2 | 4 |
| Carried up . | 289 | 276 | 565 | Totals . . . | 432 | 377 | 809 |

Dementia constituted the most common variety of mental maladies, seeing upwards of one-fourth of the insane population, or 210 persons, are enumerated under that category. Chronic mania appears next in amount, 171 cases being included in that division. Then imbecile patients, of which 92 examples are recorded; whilst there were 70 cases of lypemania, besides other varieties, although much less numerous. In addition to the particulars now detailed, it is also instructive to state that 103 individuals, or one-eighth of the aggregate number, suffered from goître along with their mental affection; 88 had enlarged necks, 57 exhibited varicose veins, 28 were both deaf and dumb, in 27 hernia existed, 14 could not hear, and lastly, 39 helpless human beings were constantly confined to bed by physical infirmities. Notwithstanding these discouraging obstacles towards ameliorating the afflicted condition of numerous inmates, in consequence of improved sanatory measures recently adopted, by abating several insalubrious influences, establishing schools, and especially the judicious application of bodily labour, great benefits have supervened. Besides such adjuvants in treating cases of mania, from the extended development of moral management, of late very perseveringly pursued at this asylum, the best results have followed: as well in reference to the physical condition of numerous inmates, as also in regard to their mental faculties. Through these appliances, the general aspect of this institution has recently become greatly ameliorated, and the lunatic's position considerably improved.

After minutely inspecting the various court-yards, dormitories, and gardens of Maréville, I can justly say, the general appearance was satisfactory, and very creditable to its executive. The tranquillity prevailing amongst so many insane persons, with their orderly conduct, attracted attention; and the bodily health of most inmates was evidently good. Numbers were employed in the gardens, and others assisted ordinary workmen in building several new constructions. Some worked at trades, handicrafts, and in household occupations; whilst the various duties necessarily required in such a large aggregate population proved a fertile source of employment. Upwards of half the patients were usually occupied; Dr. Morel being a strenuous advocate for employing lunatics in physical labour, according to their individual capabilities. Indeed, permission to work is even occasionally considered a favour granted to particular maniacs, who join other inmates, and thus gain the means of augmenting present comforts, besides contributing towards their future welfare. On the other hand, to debar such parties from joining fellow work-people is often deemed a punishment.

No farm being attached to this asylum, out-door labour is confined to the gardens adjoining, which are, however, extensive, and will be soon considerably enlarged. Further, new terraces for flowers and shrubbery, with additional gravel walks, being now in course of construction, ample opportunities are thereby afforded for employing a very large number of labourers. Besides, as various old buildings with the ancient cells, or rather dungeons, where furious lunatics were formerly immured, are also about to be demolished, in order to construct new but much improved dormitories, the extensive alterations contemplated will supply abundant employment, during many months consecutively.

Consistently with the principles actuating Dr. Morel, when treating an insane patient committed to his charge, it may be anticipated that, very little personal coercion is employed in the populous asylum of Maréville. Such is the fact; and it consequently becomes very gratifying to state, amongst the whole 471 male patients under treatment, not one was restrained in any manner. Of the 405 female lunatics also resident in the asylum, only three were under partial physical coercion; not, however, with camisoles, but simply by means of the sleeves of their ordinary gowns being tied together, which were made rather long for that purpose. By this mode, the party was prevented from tearing her own clothes, or annoying other patients, to which she happened to be predisposed. It should be likewise mentioned in explanation that, all three were nymphomaniacs, and became very easily excited on the slightest provocation. The strait-waistcoat, so common in many French asylums, is here very seldom employed; a great object kept constantly in view being to avoid any kind of personal coercion. Dr. Morel believed camisoles often exasperated patients, instead of rendering them tranquil, and hence, he considers it far preferable to adopt other methods of management. These doctrines are sound, as also confirmed by experience; and therefore cannot be too extensively disseminated, or carried over zealously into practice.

Matters were, however, very different in former years, and previous to the period when Dr. Morel became attached to the institution; nay, even so late as 1847, it was stated that, sometimes thirty lunatics might be seen physically confined in one of the ancient apartments, which now remain without a single occupant. An old dungeon—dark and dismal, was subsequently pointed out for my inspection, where dangerous maniacs were formerly placed, although often left entirely naked. These unfortunate human creatures always slept upon straw; and as the floor of this prison-like apartment was under ground, the locality seemed wholly unfit for any purpose, excepting as a store for lumber or firewood. Fortunately, the practices here recorded as characteristic of by-gone times, have now become matters of history: and are merely alluded to in these pages, in order to indicate the great advances recently accomplished in the management of lunatics at Maréville, through improved knowledge, aided by advanced civilization.

Various examples might be here quoted, to prove the efforts constantly made by Dr. Morel to discontinue restraint, wherever camisoles had been previously employed. One illustration will, however, suffice. The case was that of a male patient, who often became so excited that a strait-waistcoat seemed necessary, in the opinion of former attendants, as he was considered very mischievous, according to statements made on his arrival. Having taken proper precautions, lest any accident should happen, and after removing every ligature wherewith the maniac was confined, Dr. Morel put a pickaxe into his hand, before joining several labourers at work in the garden. Instead of attacking any bystander, as some expected, this liberated patient immediately ran towards the other workmen, and at once began to use his pickaxe assiduously, appearing at the same time delighted with such a novel occupation. Ever afterwards, although previously violent, the lunatic was often anxious to be employed; whereby he became tranquil and industrious, being altogether the reverse of his former excited condition.

Besides occupation, amusement and instruction likewise constitute essential parts of the system pursued at Maréville. Amongst the appliances frequently called into requisition to carry out these views, music—both vocal and instrumental—occupies a prominent place. Concerts are consequently held, at which patients of both sexes assist: when not only pleasure is imparted to the audience by these peformances, but beneficial results seemed often thereby produced upon individuals. Occasionally, also, parties of inmates promenade in the adjoining fields; whilst others enjoy pastoral pic-nics, held in the neighbouring forest. Respecting these recreations, it is pleasing to mention they are much appreciated by the parties partaking, and have never led to inconvenience. Again, on saints'-days, but particularly during Sundays, many patients assemble in the asylum chapel, where 700 lunatics, male and female, sometimes meet together, like any ordinary congregation. No bad consequences follow such large assemblages; indeed, the service, in which the lunatics are then engaged, frequently proves decidedly beneficial. Even dirty and epileptic patients attend on such occasions, but these inmates always occupy a side division appropriated for their special reception; so that, should untoward occurrences supervene, the general congregation shall not be disturbed in their devotions.

Having visited several refectories, while numerous parties were at dinner, besides the tranquillity then apparent, even amongst dirty or epileptic patients, and those classed as excited lunatics, it deserves being mentioned that, knives and forks were used almost universally. In one apartment I saw forty inmates at table, each having a napkin, with knife and fork, whilst all behaved like persons perfectly sane. Even many female maniacs, who were, otherwise, often noisy and talkative, if not clamorous, then sat quietly during their repast. Such marked conduct is always satisfactory; since it clearly shows that some self-control has been already acquired over individual emotions, which often smooths the path leading to subsequent improvement, if not convalescence.

In consequence of many extensive improvements now in progress, inconvenience has occasionally arisen, by crowding patients too much together in particular dormitories. This result is, however, only temporary; and will soon be amply compensated by the greater comforts, as well as improved salubrity, which these alterations must inevitably produce. Very recently, a spacious new day-room has been opened in the female division, for dirty patients and their attendants. The accommodation afforded is excellent, and certainly merits every encomium which visitors have uniformly expressed respecting this building. The apartment is large and lofty; whilst the pretty flower parterres, and an airy court-yard, with open verandahs in front, where inmates may promenade under shelter during bad weather or sunshine, are certainly great acquisitions. Besides being, in various respects, pleasing to the eye, this division which contained many female lunatics affected with very severe forms, both of mental and physical disease, was really clean in appearance; indeed, could the term be justly applied to such a receptacle of human misery, it even looked cheerful.

Notwithstanding cholera prevailed in the immediate neighbourhood of Maréville, during 1849, and proved also very fatal at Nancy, no death by that epidemic was reported within its precincts. This remarkable exemption from a malady, whereby destructive effects were often produced in various French lunatic institutions, no doubt was mainly promoted through improved regimen, and efficient sanatory measures, instituted at this asylum by the executive: which tended to counteract any prevalent epidemic influence, and hence its recent immunity.

With reference to the bodily health of patients, although many lunatics were of advanced age, and also liable to various infirmities incident to indigent persons, the general health of most residents was, on the whole, satisfactory; whilst very few actually occupied the infirmary. Amongst the individuals recently under medical treatment, irrespective of any mental affection, one patient was pointed out, whose case deserves notice, on account of its successful termination, considering the original cause which placed the party in that division. This lunatic, then labouring under religious frenzy, swallowed a metal cross usually worn by ecclesiastics. On the accident being ascertained, purgatives were exhibited, the patient being also placed under strict regimen, and carefully watched; but nothing appeared until after the lapse of fifteen days, when he voided the missing cross. Every symptom produced, by the presence of this large foreign body within the alimentary canal having ceased soon afterwards, he left the infirmary convalescent. Although the preceding case was an instance of religious madness, that variety of mental disease is not of common occurrence in this part of France, especially if compared with the neighbouring province of Alsace, where it appears to be more prevalent. Farther, examples of erotomania are likewise less frequently observed throughout Lorraine, than in more southern provinces. On the other hand, insanity produced by intoxicating drinks seem by no means rare in this district; which opinion is, unfortunately, too well-founded, seeing this asylum recently contained numerous examples of that particular form of mental malady, designated dypsomania.

According to statements given in a previous page, it may be observed that, goître frequently affects the insane patients at Maréville. This fact becomes an interesting subject of inquiry; and four crétins being likewise reported as recently resident in the asylum, a few remarks on these affections cannot be held irrelevant: especially, as both prevail to some extent in the Vosges and Meurthe departments. Although rather common in particular localities, the ratio of goîtreux complaints and crétinism appears greatest, amongst the population residing in the commune of Rosières, about twelve miles distant from Nancy. In this small town, situated at the bottom of a moderately elevated hill, in the midst of a rich fertile valley, open to the north, the east and the south, with vineyards on the west, and gypsum quarries or beds of rock-salt in its vicinity, 32 crétins and 240 goîtreux were very lately found amongst 2250 inhabitants. Hence, the proportion of crétins was 1·42 per hundred persons living in the district; and 10·66 of the latter malady, or one in every nine-and-a-third persons actually resident. It is farther worth mentioning that, in the commune of Sainte Marie-aux Mines, situated in the Vosges mountains, containing 11,000 inhabitants, 111 idiots and 60 crétins were ascertained to exist, not long ago, according to authentic documents. The facts now stated indubitably prove goître and crétinism to be common complaints throughout Lorraine, in which province both maladies have prevailed from time immemorial. Compared with other districts, crétinism seems more general in the two places just named, than throughout several countries hitherto considered peculiarly afflicted with that calamity. For instance, in the Canton de Vaud, the proportion of crétins is reported to be one case in every 463 inhabitants; and, although said to be more frequent in the Valais, it seems very doubtful if the ratio equals either that recorded at Sainte Marie-aux Mines, or Rosières.

The prevalence of goître and crétinism in the former place has been ascribed to the severity of its climate, especially to long and rigorous winters. Here, atmospheric variations are often so sudden that, extreme heat and great cold alternately occur during twenty-four hours; and as the town lies enclosed between two mountain chains, on an elevated position relative to its neighbouring plain of Alsace, there exists very little communication with adjoining districts. Instead of being robust, the labouring classes frequently seem debilitated in constitution, generally endued with lymphatic temperaments, and exhibit a scrofulous diathesis, whilst premature old age soon supervenes. It also deserves mention that, in this secluded spot—still peopled by descendants of the aborigines of Lorraine and Alsace, of refugees of the edict of Nantes, as also of German emigrants—the characteristic types of each race may be even distinguished amongst the present generation. Although some writers have attributed the existence of goître, in certain localities, chiefly to the water used by the inhabitants; to ascribe its appearance to one specific cause, is equally erroneous and unphilosophical. Defective nutrition, badly ventilated lodgings, humidity arising from confined situations, the absence of sunshine with its vivifying influence, and the constant neglect of intellectual culture, all tend to augment these complaints amongst predisposed populations. Therefore, considering the great prevalence of goître and crétinism in the Meurthe and Vosges departments, besides the Upper and Lower Rhine, it cannot appear surprising if Maréville contained four crétins last January, and so many as 105 goîtreux patients. Nevertheless, it must be highly satisfactory to know that, both these affections appear to have decreased in number, consentaneous with the advance of civilization.

During former years, goître was more common in this asylum than recently; and even cases then appeared to be actually generated within its precincts. At present, similar instances never supervene; nay, parties with large necks on entrance have occasionally exhibited considerable amelioration in the size of such glandular swellings, after residing for some time at the institution. Those results seemed mainly owing to the improved physical comforts which such individuals obtained, being altogether different from their previous indigent condition. Instead of bad food, damp, ill-ventilated houses, or miserable hovels, to speak more correctly, where they lived immured in filth, and devoid of all mental education, these unfortunate members of the great human family became inmates of an institution, whereby they were not only much better fed, but generally placed in a superior physical and moral condition. Contrasted with former privations, it is reasonable to conclude these maladies should diminish amongst residents under such circumstances. Indeed, Dr. Morel's ample experience fully confirms the correctness of every remark previously made, respecting the prevalence of goître and crétinism, throughout this part of the French republic; in which more persons are said to be afflicted by these maladies, than perhaps exists in any other country of Europe, not even excepting the Valais canton in Switzerland.

Irrespective of the numerous and varied improvements recently effected at Maréville, besides those still in progress, of which many have been greatly promoted by the director, Dr. Renemdin's administrative energy, before concluding this report, a new and important phase in the asylum must not be overlooked, viz., Dr. Morel's lectures on mental diseases, recently delivered to pupils attending the Nancy school of medicine, as also his professional friends. The course was assiduously attended by about thirty auditors on an average: and every discourse being practical, they were much appreciated; the more so, seeing the lecturer appeared deeply versed in his subject, at the same time the principles advocated were sound, whether in regard to administering lunatic asylums, or the treatment of insanity.

During the delivery of these lectures the greatest decorum invariably prevailed, even when any insane patients were introduced to the assembled audience, by way of illustrating various types of mental disease. The whole proceeding was altogether so satisfactory that, Dr. Morel merits, not only much praise in thus disseminating practical knowledge, based upon experience, respecting insanity, its symptoms, and treatment: but his name should be associated with those deservedly distinguished physicians, Ferrus, Leuret, Botex, Falret, and Baillarger, who have become honourably known for the successful efforts they have made in France to diffuse, amongst their countrymen, correct views regarding the nature, pathology, and hygiene of this important department of medical science.

(*To be continued.*)

